# The Medical Examiners Bill

**Published:** 1893-02

**Authors:** 


### *EDITORIAL.*

The office of THE JOURNAL is in room 330 Equitable Building.
Address communications relating to the Editorial Department to DR. L. B. GRANDY, Atlanta, Ga., Box 431.
Business communications should be addressed to DR. M. B. HUTCHINS, Atlanta, Ga.
All remittances should be made payable to "ATLANTA MEDICAL AND SURGICAL JOURNAL."
Articles for publication should reach this office not later than the 15th instant.
The editors are not responsible for views expressed by contributors.

### THE MEDICAL EXAMINERS BILL.

The friends of better medical laws than we now have in Georgia will be glad to know that no effort will be spared to have the bill passed by the Senate during the late session of the present legislature also favorably acted upon by the House at the coming session.

The bill was not presented to the legislature until near the close of the session. It was reported unanimously by the Senate Judiciary Committee, with the recommendation that it be passed, and received a very flattering vote in that body. It was, however, transmitted to the House too late for mature consideration; therefore at the request of the friends of the bill it was laid upon the table, to be called up after the reconvening of the legislature this fall.

In the meantime the friends of the bill should not be inactive. Every effort should be put forth in its behalf. It will be carried before the State Association, where it should be freely discussed and digested. The whole profession should become familiar with its provisions so as to be able to discuss it intelligently. Doctor, you owe it to humanity, to your profession, to yourself and to posterity to see that this or a better law be enacted. No reform is ever attempted that does not meet with opposition, but that should only enable us the better to demonstrate the justness of our cause. Ah, Doctor, how would you like for your loved ones to be left to the tender mercies of one of the horde of dishonest quacks and charlatans that now infest our State? If you saw your neighbor's house on fire would you not hasten to tell him of his danger and try to prevent the destruction of his property? Is not his life of far more value to him and his family than his house? And yet we sit calmly by and see our neighbor's loved ones, who are just as dear to him as are our loved ones to us, suffer and die from inexcusable ignorance on the part of medical attendant without raising hand or voice in their defence? Is the crime less than murder? Are not we *particeps criminis?*

The members of the legislature only need to be convinced that the measure is in the interest of humanity and not of the doctors. Perhaps you are the family physician of some one or more legislators. If so, your influence with him will be great. Will you not exert that influence as your mature judgment dictates?

No intelligent practitioner of medicine, of whatever school, should be excluded or unfairly treated. But ignorance, no matter in what guise it may appear, should be laid bare to the world. No man can be a good physician who is not liberal in all his tendencies, and every man should employ those remedies and methods of treatment which in his judgment will best conserve to the lives and health of his patients, no matter from what source the knowledge came. F. W. M.

